# Impaired Speaking-Induced Suppression in Alzheimer’s Disease

**DOI:** 10.1523/ENEURO.0056-23.2023

**Published:** 2023-06-05

**Authors:** Kyunghee X. Kim, Corby L. Dale, Kamalini G. Ranasinghe, Hardik Kothare, Alexander J. Beagle, Hannah Lerner, Danielle Mizuiri, Maria Luisa Gorno-Tempini, Keith Vossel, Srikantan S. Nagarajan, John F. Houde

**Affiliations:** 1 Department of Otolaryngology−Head and Neck Surgery, University of California San Francisco, San Francisco, CA 94117; 2 Department of Radiology and Biomedical Imaging, University of California San Francisco, San Francisco, CA 94117; 3 Department of Neurology, University of California San Francisco, San Francisco, CA 94158

**Keywords:** speaking-induced suppression, Alzheimer’s disease, state feedback control model, efference copy, magnetoencephalography

## Abstract

Alzheimer’s disease (AD) is a neurodegenerative disease involving cognitive impairment and abnormalities in speech and language. Here, we examine how AD affects the fidelity of auditory feedback predictions during speaking. We focus on the phenomenon of speaking-induced suppression (SIS), the auditory cortical responses’ suppression during auditory feedback processing. SIS is determined by subtracting the magnitude of auditory cortical responses during speaking from listening to playback of the same speech. Our state feedback control (SFC) model of speech motor control explains SIS as arising from the onset of auditory feedback matching a prediction of that feedback onset during speaking, a prediction that is absent during passive listening to playback of the auditory feedback. Our model hypothesizes that the auditory cortical response to auditory feedback reflects the mismatch with the prediction: small during speaking, large during listening, with the difference being SIS. Normally, during speaking, auditory feedback matches its predictions, then SIS will be large. Any reductions in SIS will indicate inaccuracy in auditory feedback prediction not matching the actual feedback. We investigated SIS in AD patients [*n *=* *20; mean (SD) age, 60.77 (10.04); female (%), 55.00] and healthy controls [*n *=* *12; mean (SD) age, 63.68 (6.07); female (%), 83.33] through magnetoencephalography (MEG)-based functional imaging. We found a significant reduction in SIS at ∼100 ms in AD patients compared with healthy controls (linear mixed effects model, *F*_(1,57.5)_ = 6.849, *p *=* *0.011). The results suggest that AD patients generate inaccurate auditory feedback predictions, contributing to abnormalities in AD speech.

## Significance Statement

Speaking-induced suppression (SIS) refers to the suppressed response to auditory feedback heard during speaking, compared with the response to the same feedback heard during passive listening. We posit that SIS arises from a comparison between auditory feedback and a motor-efference-copy-derived feedback prediction. During speaking, feedback matches prediction, resulting in small auditory responses. Passive listening to playback of this feedback matches no prediction, resulting in large auditory responses. Thus, SIS measures the accuracy of feedback predictions. The absence of SIS that we found in Alzheimer’s disease (AD) patients suggests they have impaired feedback predictions, which may be indicative of a more widespread impairment in generating predictions that may be manifested in the earliest stages of AD progression.

## Introduction

Abnormalities in speech production in Alzheimer’s disease (AD) have received scant attention in the literature. However, AD patients exhibit anatomic abnormalities in the cortical network associated with speech motor control, comprising superior temporal, posterior parietal, premotor, and prefrontal regions. For instance, AD patients show degeneration of a posterior parietal network (Rabinovici et al., 2007), volume decrease in dorsolateral prefrontal cortex (Baron et al., 2001; Frisoni et al., 2002; Busatto et al., 2003; Grossman et al., 2004; Ishii et al., 2005; Kawachi et al., 2006; Singh et al., 2006; Du et al., 2007), and distinct temporal lobe atrophy patterns (Chan et al., 2001), as well as speech and language impairments (Klimova et al., 2015; Szatloczki et al., 2015). Some researchers have attempted to link changes in language abilities to cognitive decline in AD (Mueller et al., 2018). Several linguistic variables have been used to predict the onset of AD (Eyigoz et al., 2020). While these independently conducted assessments with speech and language components can be beneficial for identifying early stages of AD, neurophysiological evidence of neural dysfunction during speaking (Ranasinghe et al., 2019) may provide a more sensitive prognostic measure of disease progression in AD.

The brain network associated with speaking is complex because speech motor control is a complex process consisting of feedforward and feedback control. It entails the preparation and execution of speech motor programs (feedforward control), as well as the monitoring and compensatory responses to sensory feedback fluctuations during speaking (feedback control; Findlay et al., 2012; Chang et al., 2013; Herman et al., 2013; Niziolek et al., 2013; Kort et al., 2016). We previously have shown that AD patients have abnormally large compensatory responses to perturbations of the pitch of their auditory speech feedback (Ranasinghe et al., 2017), perhaps indicating greater reliance on feedback control (Parrell et al., 2017). We also see abnormally large responses to pitch feedback perturbations in patients with cerebellar degeneration, where a greater reliance on feedback control would be consistent with impairment of the cerebellum, which is thought to play a key role (feedback prediction) in the feedforward control of movement (Parrell et al., 2017; Houde et al., 2019). It is plausible, therefore, that in AD patients, a greater reliance on feedback control may also be because of impaired feedforward control, possibly because of unreliable feedback predictions.

This need for feedback predictions in the control of speech is a key part of our state feedback control (SFC) model of speech motor control (Houde and Nagarajan, 2011; Houde and Chang, 2015). This model assumes that, while speaking, incoming auditory feedback is compared with auditory predictions that are derived from efference copy of the motor commands driving speech output. Any mismatch between feedback and prediction results in compensatory motor responses that reduce the mismatch. Thus, during speaking, the onset of speech feedback in auditory cortex is predicted from motor efference copy, creating a minimal mismatch with the feedback prediction, resulting in a small auditory cortical response. In contrast, during passive listening to speech, the unavailability of precise predictions results in a more pronounced mismatch and a large auditory cortical response. Thus, better suppression during speaking signifies good predictions, while smaller suppression implies inaccurate predictions. By comparing auditory cortical responses to self-produced speech with those obtained during its playback, a measure of speaking-induced suppression (SIS) may be obtained. SIS indexes how accurately feedback predictions match incoming auditory feedback (Niziolek et al., 2013). A reduced SIS in AD patients compared with healthy controls would support the hypothesis that internal prediction mechanisms underlying speech motor control are faulty in AD. In this study, we tested this hypothesis by examining the SIS phenomenon in AD patients and controls using magnetoencephalography (MEG). Specifically, we compared the magnitudes of auditory cortical response around 50 ms (M50), 100 ms (M100), and 200 ms (M200), following speech stimulus onset during speaking and listening to playback of the same stimulus. Low-level sensory deficits arising from primary and higher order auditory cortices, as reflected in abnormal short latency responses (M50–M200), may suggest impairments in making feedback predictions that reflect more widespread prediction deficits impacting not only speech and language perception but also other cognitive abilities (Ranasinghe et al., 2017).

## Materials and Methods

### Participants

All participants (20 AD patients and 12 age-matched controls) were recruited from research cohorts at the University of California San Francisco (UCSF) Memory and Aging Center. AD patients received a complete clinical evaluation and structural brain imaging. Patients with other dementia co-pathologies, systemic medical illnesses, or those on medications impacting central nervous system function were excluded. The eligibility criteria for age-matched controls included normal performance on cognitive tests, normal structural brain imaging, a negative Aβ-PET, the absence of a crucial cognitive decline during the previous year, neurologic or psychiatric illness, and other major medical illnesses. A structural magnetic resonance image was obtained for each participant. The participants had normal hearing except for age-related high-frequency hearing loss. Moreover, the participants or their assigned surrogate decision-makers signed informed consent. The UCSF Institutional Review Board for Human Research approved all experimental procedures.

### Neuropsychological assessment

Each participant underwent a structured caregiver interview to determine Clinical Dementia Rating (CDR) and CDR Sum of Boxes (CDR-SOB; Morris, 1993) and was assessed by Mini-Mental State Examination (MMSE; Folstein et al., 1975). Statistical tests comparing demographic characteristics and cognitive abilities for AD patients and healthy controls were conducted using SAS 9.4 (SAS Institute Inc). Patients included in this study were in the early stages of their disease depending on CDR, CDR-SOB, and MMSE scores (see Table 1).

**Table 1:** Participant demographics

	AD (*n *=* *20)	Control (*n *=* *12)	*p*-value*
Age (years)	60.77 ± 10.04	63.68 ± 6.07	0.156
Female sex, *n* (%)	11 (55.00)	10 (83.33)	0.139
White race, *n* (%)†	20 (100.00)	12 (100.00)	1.000
Education (years)	16.05 ± 2.33	17.83 ± 1.40	0.029
Right handedness, *n* (%)	17 (85.00)	12 (100.00)	0.274
MMSE‡	23.00 ± 4.34	29.67 ± 0.65	<0.00001
CDR	0.83 ± 0.41	0.13 ± 0.31	0.00016
CDR-SOB	4.23 ± 2.16	0.46 ± 1.30	<0.00001

Values for age, education, MMSE, CDR, and CDR-SOB are expressed as mean ± SD. The ages were between 49.0 and 84.0 for AD patients and 56.0 and 75.6 for healthy controls.

*Statistical testing was conducted using the Mann–Whitney *U* test for age, education, MMSE, CDR, and CDR-SOB; Fisher’s exact test for sex, race, and handedness.

†Race was self-reported.

‡The MMSE scores denote better cognitive function with higher scores in the range of 0−30.

### Experimental design

The MEG experiment comprised four blocks of 74 trials each, with ∼2.5 s per trial. In the Speak condition (blocks 1 and 3), participants were instructed to phonate the “ah” sound when a dot appeared on the projection screen and terminate phonation on arrival of a visual cue to stop. As they spoke, participants heard their own auditory speech feedback in real-time through headphones. After completing a Speak condition block, a Listen condition (blocks 2 and 4) followed. During the Listen condition, participants heard a playback of the auditory feedback they heard during the preceding Speak condition block, allowing isolation of speaking-specific activity. Breaks were provided after every 15 trials and the duration of each break was the participant’s choice.

A 275-channel whole-head MEG system (Omega 2000, CTF; sampling rate, 1200 Hz; filtering, 0.001−300 Hz) recorded neurophysiological responses from participants during the experiment. Each participant lays supine with their head supported near the center of the sensor array with three localizer coils affixed to the nasion and bilateral preauricular points to determine head positioning relative to the sensor array. Head movement was measured via difference in coil locations relative to the sensor array before and after each block of trials. If movement exceeded 7 mm, the block was rerun. Auditory stimuli were delivered to participants at comfortable levels via MEG-compatible earplugs (EAR-3A, Etymotic Research), with amplitudes comparable to side-tone levels during speaking. The amplitude of the auditory stimuli in the Listen condition was identical to that of the Speak condition. Participants produced speech responses via an MEG-compatible optical microphone (Phone-Or Ltd). Visual cues to start and stop phonation were presented against a black background at the center of a projection screen situated ∼24 inches away from the participant’s face. All stimulus and response events were integrated with MEG traces via analog-to-digital inputs in real-time using the imaging acquisition software.

Coregistration of MEG data to individual MRI images was performed using the CTF software suite (MISL Ltd.; ctfmeg.com; version 5.2.1) by aligning the three fiducial locations of nasion, left, and right peri-auricular points on the individual’s MRI (3T, Siemens) with the corresponding coil positions placed during MEG collection, after which a single sphere head model was created. Then, the MRI was exported to Analyze format and warped to the standard T1 Montreal Neurologic Institute (MNI) template via Statistical Parametric Mapping (SPM8, Wellcome Trust Centre for Neuroimaging, London, United Kingdom).

### MEG data preprocessing

Condition-specific blocks were combined to create separate Speak and Listen MEG datasets for each participant. Twenty-nine reference sensors were used to correct distant magnetic field disturbance by calculating a synthetic third order gradiometer (Weinberg et al., 1984; Vrba and Robinson, 2001), and a dual signal subspace projection algorithm was applied to eliminate speech movement artifacts in biomagnetic measurements (Sekihara et al., 2016; Cai et al., 2019). The MEG data were then filtered using a 2 Hz high-pass filter to remove slow fluctuation and marked at voice onset. Trials were segmented −300 ms to +300 ms around phonation onset, corrected using DC-offset, and filtered from 2 − 150 Hz. Trials were rejected for artifacts if MEG sensor channels exceeded a threshold value of 1.5 pT, or speech was detected during Listen trials, with manual verification of all flagged artifacts. Data from seven AD patients (*n *=* *7/27) and three healthy controls (*n *=* *3/15) were omitted from further analysis, as fewer than 50 trials remained in a condition after artifact rejection. For artifact-free data (20 AD patients; 12 healthy controls), trials were averaged to produce a single timeseries per condition, and split into separate left and right hemisphere sensor arrays to capture the auditory response from each hemisphere.

### Source reconstruction and auditory response

**Figure 1.**
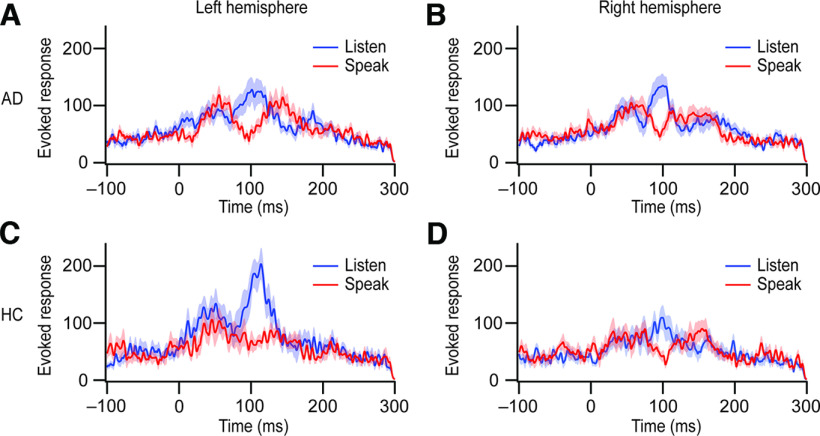
Auditory cortical time course in both the speaking and listening conditions. MEG traces were aligned to the voice onset. Thick lines denote means, and the shaded regions behind the lines denote SEM (blue, the listening condition; red, the speaking condition). ***A***, ***B***, AD patients’ mean responses (*n *=* *20) for the left hemisphere (***A***) and the right hemisphere (***B***) are depicted. ***C***, ***D***, The mean responses for healthy controls (HC; *n *=* *12) are shown in the left hemisphere (***C***) and the right hemisphere (***D***).

**Figure 2.**
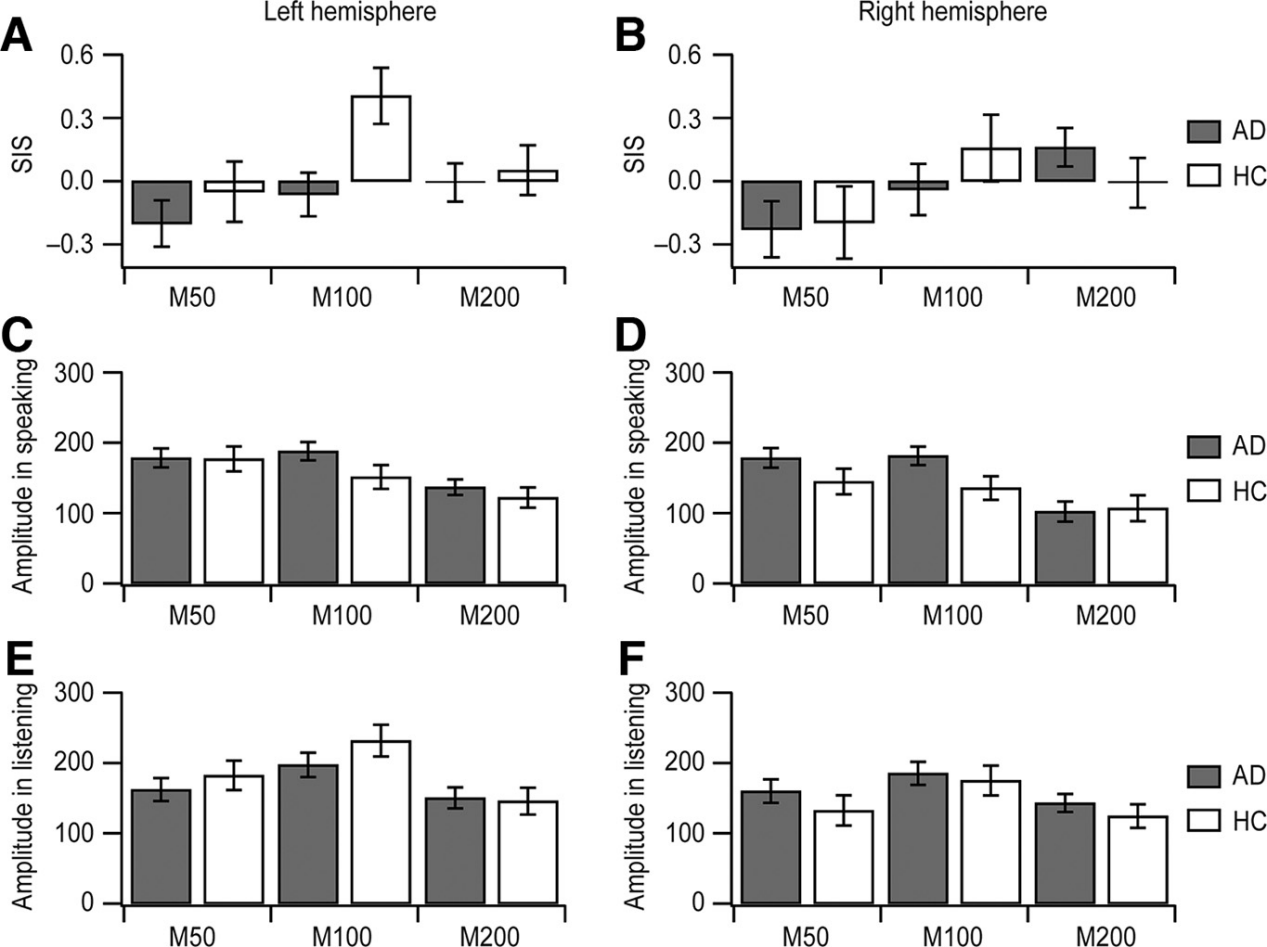
Amplitudes at M50, M100, and M200 in auditory cortical time course. AD patients’ data (AD; *n *=* *20; mean ± SEM) are presented in gray. Healthy controls’ data (HC; *n *=* *12; mean ± SEM) are in white. ***A***, ***B***, The means of individual SIS magnitudes at each peak are exhibited in the left (***A***) and right (***B***) hemispheres. ***C***, ***D***, The means of amplitudes at the three peaks from the cortical activity during speaking are depicted in the left (***C***) and right (***D***) hemispheres. ***E***, ***F***, The mean peak values of cortical responses during listening are displayed in the left (***E***) and right (***F***) hemispheres.

**Figure 3.**
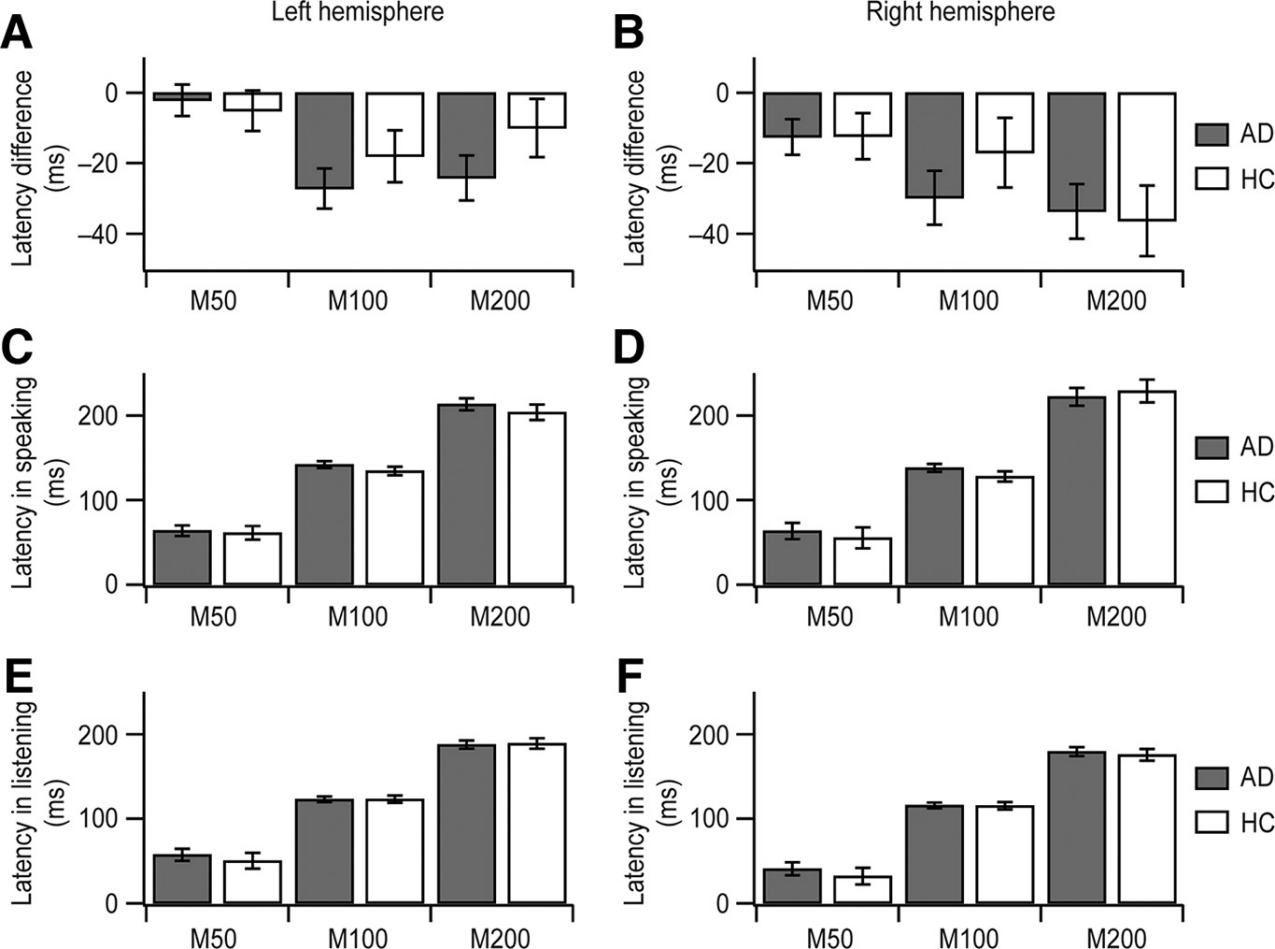
Latencies at M50, M100, and M200 in auditory cortical time course. AD patients’ data (AD; *n *=* *20; mean ± SEM) and healthy controls’ data (HC; *n *=* *12; mean ± SEM) are gray and white, respectively. ***A***, ***C***, ***E***, In the left hemisphere, the average latencies are depicted in the speaking condition (***C***), the listening condition (***E***), and the latency difference between the two conditions (***A***). ***B***, ***D***, ***F***, In the right hemisphere, the average latencies are shown in the speaking condition (***D***), the listening condition (***F***), and the latency difference between the two conditions (***B***).

Individual trial-averaged data for left and right sensor array locations underwent Bayesian covariance beamforming (Vrba and Robinson, 2002; Cai et al., 2021) focused on the MNI coordinates (left hemisphere: −54.3, −26.5, 11.6; right hemisphere: 54.4, −26.7, 11.7) linked to the primary auditory cortex in the corresponding hemisphere (Kort et al., 2014). The resulting source timeseries was transformed into root mean square (RMS) activity for each time point, yielding a timeseries of positive-going evoked activity from within the voxel nearest each MNI coordinate for the primary auditory cortex (Fig. 1). Maximal amplitude values (Fig. 2) and their corresponding latencies (Fig. 3) around the M50, M100, and M200 sensory peaks in each hemisphere were extracted from individual timeseries using a semiautomated process. First, each timeseries from the left or right primary auditory cortex was averaged across all participants, from which a latency window around the maximum deflection of each peak was defined (±20 ms for M50, ±50 ms for M100, ±50 ms for M200). Then, these windows were used to identify peak amplitudes and latencies for each individual timeseries (participant × condition) within each of the three sensory component windows. Next, extracted peaks and their latencies were visually confirmed and adjusted when necessary. Finally, SIS was calculated from each of these values: the ratio of the difference between peak amplitude in the Listen and Speak conditions, divided by the amplitude during the Listen condition (i.e., [Listen – Speak]/Listen). Subtracted peak latencies (Listen − Speak) examined differences in latency of the peak for each sensory component.

### Statistics

The distributions of peak latencies, amplitudes, and SIS values were examined for normality via Kolmogorov–Smirnov tests and then transformed using a two-step algorithm (Templeton, 2011) when warranted, before statistical analyses. Linear mixed effects modeling (IBM SPSS Statistics, version 28) was employed to explore group-related (AD vs control) significant differences in the amplitude, latency, and SIS response relative to the three peak components (PEAK), two hemispheres (HEMI) and, for nonsubtracted measures, the speaking or listening condition (COND). The model included fixed factors of GROUP and its interactions (GROUP × PEAK, GROUP × HEMI, GROUP × PEAK × HEMI), with PARTICIPANT included as a random factor and repeated factors specified as PEAK, HEMISPHERE, and PARTICIPANT. Condition appeared within the fixed interaction terms (GROUP × COND, GROUP × HEMI × COND, GROUP × PEAK × COND, GROUP × PEAK × HEMI × COND) and as a repeated factor in models where peak amplitude (not SIS) was the dependent variable. Model intercepts were included in both fixed and random effect terms. Significance was assessed at *p *<* *0.05.

## Results

### Demographic characteristics of participants

AD patients exhibited mild disease with a CDR of 0.83 ± 0.41 (mean ± SD), CDR-SOB of 4.23 ± 2.16 (mean ± SD), and MMSE of 23.00 ± 4.34 (mean ± SD). Healthy controls had similar age, sex, race, and right-handedness percentages; however, they were more educated than AD patients (Table 1).

### Time course of auditory cortical activity during speaking and listening

To examine SIS in AD, we contrasted evoked auditory responses in the speaking and listening conditions between AD patients and healthy controls. Individual evoked response power timeseries were extracted from regions of interest in the primary auditory cortex (Kort et al., 2014) for each condition and hemisphere. The group average of each timeseries is presented in Figure 1, where expected peaks representing M50, M100, and M200 are observed.

### Peak latency of auditory cortical activity

Group differences in peak latency between AD patients and healthy controls (Fig. 3*C–F*) varied across the three peaks (GROUP × PEAK, *F*_(4,180.6)_ = 320.142, *p *<* *0.001). However, this effect was largely because of differences in the within-group latency pattern across peaks rather than a between-group difference at particular peaks (GROUP; for M50, *F*_(1,106.5)_ = 1.095, *p *=* *0.298; for M100, *F*_(1,30.1)_ = 1.474, *p *=* *0.234; for M200, *F*_(1,38.2)_ = 0.023, *p *=* *0.879).

To examine the temporal relationship between the placement of peaks during speaking and listening conditions, we subtracted peak latency of the speaking condition from that of the listening condition for each peak component (Table 2; Fig. 3*A*,*B*). The negative latency differences, occurring across all groups, peak components, and hemispheres, support an overall delay in peak activity during speaking relative to listening condition. Group differences in this temporal delay occurred across peaks (GROUP × PEAK, *F*_(4,96.6)_ = 6.149, *p *<* *0.001) and, as with nonsubtracted latencies, reflected a different within-group pattern across peaks rather than differences between groups at the individual M50, M100, or M200 components.

**Table 2:** Speaking-induced suppression and peak latency difference between the speaking and listening conditions

	Patients with Alzheimer’s disease (*n *=* *20, mean ± SEM)		Healthy controls (*n *=* *12, mean ± SEM)	
	Left hemisphere	Right hemisphere	Left hemisphere	Right hemisphere
SIS, M50	−0.20 ± 0.11	−0.23 ± 0.13	−0.05 ± 0.14	−0.20 ± 0.17
SIS, M100	−0.06 ± 0.10	−0.04 ± 0.12	0.41 ± 0.13	0.16 ± 0.16
SIS, M200	−0.01 ± 0.09	0.16 ± 0.09	0.05 ± 0.12	−0.01 ± 0.12
Latency diff, M50 (ms)	−2.2 ± 4.5	−12.5 ± 5.1	−5.1 ± 5.8	−12.3 ± 6.5
Latency diff, M100 (ms)	−27.2 ± 5.7	−29.8 ± 7.6	−18.0 ± 7.4	−17.0 ± 9.9
Latency diff, M200 (ms)	−24.2 ± 6.4	−33.7 ± 7.8	−10.0 ± 8.3	−36.3 ± 10.0

Latency diff = peak latency difference between the speaking and listening conditions.

### Impaired speaking-induced suppression in Alzheimer’s disease

We then analyzed group differences in peak amplitude for the speaking and listening conditions (Fig. 2*C–F*). While no overall main effect of GROUP was observed in the peak amplitude, significant interactions with GROUP were observed with peak component (GROUP × PEAK, *F*_(4,212.8)_ = 16.999, *p *<* *0.001), hemisphere (GROUP × HEMI, *F*_(2,295.8)_ = 7.137, *p *<* *0.001), and speaking/listening condition (GROUP × COND, *F*_(2,295.8)_ = 4.247, *p *=* *0.015), as well as a three-way interaction with condition and peak component (GROUP × PEAK × COND, *F*_(4,212.8)_ = 3.610, *p *=* *0.007).

Further analyses within each of the three component peaks revealed that amplitude was, across conditions, not significantly different between the two participant groups (GROUP; for M50, *F*_(1,29.6)_ = 0.437, *p *=* *0.514; for M100, *F*_(1,33.4)_ = 0.662, *p *=* *0.422; for M200, *F*_(1,31.0)_ = 0.288, *p *= 0.595). However, significant GROUP × CONDITION interactions were obtained for the M100 (*F*_(2,81.1)_ = 6.406, *p *= 0.003) and M200 (*F*_(2,85.0)_ = 4.362, *p *=* *0.016) peaks, but not for M50 (*F*_(2,88.7)_ = 0.935, *p *=* *0.396). *Post hoc* analyses separating speaking and listening conditions revealed a significant group difference only for M100 during the speaking condition (*F*_(1,30)_ = 6.222, *p *=* *0.018), but not during the listening condition (*F*_(1,30) _= 0.269, *p *=* *0.608). Group differences were not obtained in either condition during the M200 period (speaking: *F*_(1,30)_ = 0.104, *p *=* *0.750; listening: *F*_(1,30)_ = 0.373, *p *=* *0.546). Hence, we observed significant group effects for M100 amplitude during speaking for the patient group compared with healthy participants. No substantial M100 amplitude reduction during speaking was apparent in AD patients (Figs. 1*A*,*B*, 2*C*,*D*). We directly compare this effect at the M100 peak component using the SIS measure below.

To determine whether SIS amplitudes in AD differ from SIS amplitudes in healthy controls, the amplitude difference in auditory cortical responses was analyzed using SIS. Consistent with the findings above, a group difference in SIS was found across the three peaks of M50, M100, and M200 (GROUP × PEAK, *F*_(4,111.8)_ = 4.245, *p *=* *0.003), again with no hemispheric difference between AD patients and healthy controls (GROUP × HEMI, *F*_(2,139.3)_ = 1.189, *p *=* *0.308). *Post hoc* tests confirmed that reduced SIS for AD patients relative to healthy age-matched participants occurred specifically at M100 (GROUP; for M50, *F*_(1,30.0)_ = 0.326, *p *=* *0.573; for M100, *F*_(1,57.5)_ = 6.849, *p *=* *0.011; for M200, *F*_(1,30.0)_ = 0.259, *p *=* *0.615). In healthy controls, SIS was extant at M100 in both hemispheres (Fig. 2*A*,*B*; Table 2), confirming previous findings that SIS originated primarily from M100 responses (Houde et al., 2002; Ventura et al., 2009; Niziolek et al., 2013; Kort et al., 2014). In the left hemisphere, SIS values at M100 were −0.06 ± 0.10 (mean ± SEM) for AD patients and 0.41 ± 0.13 (mean ± SEM) for healthy controls (Fig. 2*A*; Table 2). Likewise, in the right hemisphere, healthy controls had a substantially higher SIS than AD patients [AD patients, −0.04 ± 0.12 (mean ± SEM); healthy controls, 0.16 ± 0.16 (mean ± SEM); Fig. 2*B*; Table 2]. A diminished SIS in AD patients appears to be because of the substantial contribution of unsuppressed peak amplitudes at M100 during speaking, rather than the impact of decreased peak amplitudes at M100 during listening (see above).

## Discussion

This is the first study to demonstrate that SIS of the M100 responses from the auditory cortex is absent in AD patients, while SIS is evident in matched older adult controls. The reduced SIS in AD patients compared with healthy controls supports the hypothesis that internal prediction mechanisms underlying speech motor control are faulty in AD. Below, we discuss why impaired auditory feedback prediction processes would lead to a reduction in SIS. The absence of SIS that we found in AD patients suggests they have impaired feedback predictions which may be indicative of a more widespread impairment in generating predictions that may be manifested in the earliest stages of AD progression.

Speakers appear to monitor their sensory feedback during speaking, comparing incoming feedback with feedback predictions, a process that is predominantly automatic, unconscious and prospective (Houde et al., 2002; Hickok et al., 2011; Houde and Nagarajan, 2011; Ford and Mathalon, 2012; Chang et al., 2013; Kort et al., 2014). Speakers experience self-agency only when auditory feedback minimally deviates from predicting what they expect to hear (Korzyukov et al., 2017; Subramaniam et al., 2018). When speakers hear minimal perturbations of their auditory feedback while speaking, they typically make compensatory corrective responses that oppose the perturbation direction, showing that they judge the perturbations to be errors in their speech output (Houde et al., 2002; Houde and Nagarajan, 2011; Chang et al., 2013; Kort et al., 2014; Ranasinghe et al., 2017).

These compensatory responses to feedback perturbations are accounted for in our SFC model of speech motor control. In the SFC model, the state of the vocal tract articulators is continually being estimated during speaking. The estimated state is compared with the desired state of the articulators appropriate for the current speech sound being produced, and controls are issued to the vocal tract to make the estimated state track the desired state. The current articulatory state is estimated via a prediction/correction process, where the next state of the articulators is first predicted from the previous estimate and efference copy of the controls currently being issued to the vocal tract. This state prediction is then used to predict the current sensory feedback expected from the vocal tract. Incoming sensory feedback is compared with these predictions, and any prediction errors are converted to corrections to the predicted state, resulting in an updated estimate of the current articulatory state. If the updated state estimate differs from the current desired state, controls are issued to the vocal tract, generating a compensatory response.

Thus, in the auditory cortex, the SFC model supposes that, during speaking, incoming auditory feedback is compared with efference-copy derived predictions of that feedback, and measuring SIS should provide an index of the accuracy of the auditory feedback predictions. The above discussion also suggests that inaccurate predictions would lead to large prediction errors, causing large state corrections and ultimately leading to large compensatory responses. In this way, the abnormal SIS in AD patients is consistent with the abnormally large compensations in their response to auditory perturbations. Low-level sensory deficits arising from primary and higher order auditory cortices, as reflected in abnormal short latency responses (M50–M200), may suggest impairments in making feedback predictions. Predictive processes have been posited to be fundamental components of models of various cognitive domains (Bubic et al., 2010). Low-level deficits found here may reflect more widespread prediction deficits impacting not only speech and language perception but also other cognitive abilities (Ranasinghe et al., 2017). Thus, we believe the abnormalities in speech motor control reveals core deficits that may be manifested in the earliest stages of AD progression, and may be sensitive biomarkers of the disease pending further evidence in correlative longitudinal clinical and imaging studies of AD progression.

Although the hemispheric difference in SIS amplitudes between AD patients and healthy controls did not reach statistical significance (see Results), SIS at M100 was higher in the left hemisphere in controls (Fig. 2*A*,*B*; Table 2). The left hemisphere dominance of SIS in healthy participants also aligns with our SFC model (Houde and Nagarajan, 2011; Houde and Chang, 2015), which proposes that the left hemisphere primarily detects auditory feedback prediction errors, whereas the right hemisphere converts these errors into state corrections (Tourville et al., 2008; Kort et al., 2013).

Previous studies have established that AD patients have overactivity in the posterior temporal lobe (pTL) and underactivity in the medial prefrontal cortex (mPFC). These are associated with abnormally large responses to pitch perturbations (Ranasinghe et al., 2017, 2019). The degree of compensation and mPFC activity during compensation are also correlated with measures of cognitive abilities in AD patients, particularly those of executive function (Ranasinghe et al., 2019). These findings are particularly important because other studies have shown evidence that activity in mPFC appears to index confidence in the accuracy of the prediction of what subjects expect to hear when they speak. Several previous studies have shown that activity within mPFC correlates with successful self-predictions (Subramaniam et al., 2012, 2018; Franken et al., 2018; Khalighinejad et al., 2018), indicating a neural correlate of self-agency. High confidence in prediction accuracy is reflected in high mPFC activity, while low confidence is reflected in lower mPFC activity. Our SFC model says that mPFC inhibits the state correction process in pTL that ultimately drives perturbation responses. Thus, in AD, underactivity in mPFC would disinhibit pTL, resulting in overactivity in pTL and large perturbation responses. If the auditory feedback predictions of AD patients were more variable and inaccurate, this would result in underactivity of mPFC. In this way, our finding that AD patients are compromised at predicting auditory feedback could also account for low activity in mPFC in AD during speech production.

To finish our discussion, we would like to acknowledge some limitations of this study. The first limitation is the smaller sample size, which could affect the results’ reliability by reducing the power of the study and increasing the margin of error. However, this study’s sample size is similar to previous studies involving AD patients (Ranasinghe et al., 2017, 2019) with consistent results. Another limitation is that we used an MNI template brain for specifying the primary auditory cortex based on the anatomic atlases, which was reverse transformed into the individual’s MRI coordinates for source reconstruction. It may be better to use a cohort specific (AD or older adult controls) template for the reduction of anatomic variability in our cohorts. Furthermore, it is possible that the functional location of the auditory cortex is variable across subjects independent of the anatomy. Instead of specifying the location of the auditory cortex anatomically, an alternative approach would be functional specification of the auditory cortex in individual subjects, for example by localizing the auditory evoked field response to simple tones in each participant’s brain (Ventura et al., 2009).

In summary, this study discovered abnormalities in speech motor control in AD patients, characterizing their reduced SIS. Our SFC model of speech motor control suggests that the diminished SIS is consistent with the impaired auditory feedback predictions in AD, contributing to generating overly large compensatory changes in articulatory controls. Uncovering the specific patterns of speech-motor-control network dysfunctions relating to early speech and language impairments in AD will enable us to identify some of the earliest network abnormalities in this disease.

## Synthesis

Reviewing Editor: Christoph Michel, Universite de Geneve Decisions are customarily a result of the Reviewing Editor and the peer reviewers coming together and discussing their recommendations until a consensus is reached. When revisions are invited, a fact-based synthesis statement explaining their decision and outlining what is needed to prepare a revision will be listed below. The following reviewer(s) agreed to reveal their identity: Benedikt Zoefel. Note: If this manuscript was transferred from JNeurosci and a decision was made to accept the manuscript without peer review, a brief statement to this effect will instead be what is listed below.

The reviewer is happy with the answers to the comments and the revision of the manuscript and recommends acceptance.
